# Chronic Pain: New Insights in Molecular and Cellular Mechanisms

**DOI:** 10.1155/2015/676725

**Published:** 2015-03-23

**Authors:** Livio Luongo, Marzia Malcangio, Daniela Salvemini, Katarzyna Starowicz

**Affiliations:** ^1^Department of Experimental Medicine, Division of Pharmacology, Second University of Naples, 80138 Naples, Italy; ^2^Wolfson Centre for Age Related Diseases, King's College London, Guy's Campus, London SE1 1UL, UK; ^3^Saint Louis University School of Medicine, Saint Louis, MO 63104, USA; ^4^Laboratory of Pain Pathophysiology, Department of Pain Pharmacology, Institute of Pharmacology, Polish Academy of Sciences, UL Smetna 12, Krakow, Poland

Chronic pain such as neuropathic pain, osteoarthritic pain, or abnormal pain associated with neurological diseases represents a debilitating condition which strongly affects the quality of life of patients. The mechanisms at the basis of the induction and maintenance of chronic pain are still poorly understood. Thus, an appropriate therapy for chronic pain is not yet available and there are many failures in treatments.

Recent evidence suggests a role for central and peripheral immune cells (microglia, macrophages, astrocytes, mast cells, and T-cells) in the initiation of peripheral and central sensitization. They mediate the plastic changes occurring within pain pathways that result in sensory dysfunctions and behavioural correlates, such as thermal/mechanical hyperalgesia and tactile allodynia. Because of the complex molecular and cellular mechanisms involved in the neuropathic pain induction and maintenance, several mediators have been demonstrated to be crucial in its induction and maintenance in the last years.

Historically the NMDA receptor for glutamate has been deeply investigated for the spinal wind-up occurring in the establishment of tactile allodynia. The role of the NMDA NR2B subunit as well as a possible pharmacological activity of its natural agonist, the D-aspartic acid, has been further clarified in this issue. More recent data suggest a role for lipid-mediated pathways such as sphingosine-1-phosphate or endocannabinoid systems in the modulation of spinal and supraspinal events associated with peripheral neuropathy [1, 2]. Besides the endocannabinoids also the “so-called” endocannabinoid-like molecules such as palmitoylethanolamide and oleoylethanolamide (PEA and OEA) have been demonstrated to be potentially useful to treat neuropathic pain-associated allodynia and hyperalgesia [3–5].

In this issue further investigations on the possible alterations in the anandamide metabolism in the development of neuropathic pain have been addressed. Moreover, the possible use of the palmitoylethanolamide in the delay of morphine tolerance has also been suggested. In this special issue, focused on further understanding of the molecular and cellular mechanisms at the basis of the chronic neuropathic pain, several other neuromodulatory systems have been analyzed. A possible role of a new class of chemokines, the prokineticins, of the somatostatin receptor activation, of the nociceptin/orphanin, and of the mTOR pathway in neuropathic pain mechanisms has been further elucidated. Moreover, the possible use of a still far stem cells therapy has been reviewed in the issue.

Nowadays a pivotal role of microglia in the establishment of tactile allodynia is confirmed. The microglia activation and recruitment seem to be highly regulated by purinergic system. Indeed, it has been highlighted that the abnormal ATP release in the dorsal horn of the spinal cord is responsible for the BDNF release from microglia through a mechanism mediated by P_2_X_4_ which, in turn, causes the shift in neuronal anion gradient [6]. Moreover, also a role for the P_2_X_7_ in the microglial release of IL-1*β* and other proinflammatory molecules such as cathepsin S has been demonstrated [7]. The role of the purinergic system in the regulation of glial and microglial cells has been extensively reviewed in the paper by G. Magni and S. Ceruti.

In conclusion, we hope that the readers will find in this special issue a discreet panoramic view of the puzzling mechanisms involved in chronic pain development.

*Livio Luongo* *Marzia Malcangio* *Daniela Salvemini* *Katarzyna Starowicz*
